# PhenoScanner: a database of human genotype–phenotype associations

**DOI:** 10.1093/bioinformatics/btw373

**Published:** 2016-06-17

**Authors:** James R. Staley, James Blackshaw, Mihir A. Kamat, Steve Ellis, Praveen Surendran, Benjamin B. Sun, Dirk S. Paul, Daniel Freitag, Stephen Burgess, John Danesh, Robin Young, Adam S. Butterworth

**Affiliations:** ^1^ Cardiovascular Epidemiology Unit, Department of Public Health and Primary Care, University of Cambridge, Cambridge CB1 8RN, UK; ^2^ Wellcome Trust Sanger Institute, Hinxton CB10 1SA, UK; ^3^ NIHR Blood and Transplant Research Unit, Department of Public Health and Primary Care, University of Cambridge, Cambridge CB1 8RN, UK; ^4^ Robertson Centre for Biostatistics, University of Glasgow, Glasgow G12 8QQ, UK

## Abstract

**Summary:** PhenoScanner is a curated database of publicly available results from large-scale genetic association studies. This tool aims to facilitate ‘phenome scans’, the cross-referencing of genetic variants with many phenotypes, to help aid understanding of disease pathways and biology. The database currently contains over 350 million association results and over 10 million unique genetic variants, mostly single nucleotide polymorphisms. It is accompanied by a web-based tool that queries the database for associations with user-specified variants, providing results according to the same effect and non-effect alleles for each input variant. The tool provides the option of searching for trait associations with proxies of the input variants, calculated using the European samples from 1000 Genomes and Hapmap.

**Availability and Implementation:** PhenoScanner is available at www.phenoscanner.medschl.cam.ac.uk.

**Contact:** jrs95@medschl.cam.ac.uk

**Supplementary information:** Supplementary data are available at *Bioinformatics* online.

## 1 Introduction

Genome-wide association studies (GWAS) have discovered thousands of associations between genetic variants and a wide range of human phenotypes, yielding novel insights into disease aetiology. However, a key challenge for the human genomics community is to develop methods that enable efficient cross-referencing of a genetic variant with a wide range of phenotypes, such as disease states, physiological parameters, cellular traits and other characteristics. Such ‘phenome scans’ could help inform a range of analyses, such as Mendelian randomization analyses, in which genetic variants are used as proxies for modifiable risk factors to attempt to infer causality between traits and diseases (Burgess and Thompson, 2015). Identifying the broad phenotypic consequences of perturbing a particular pathway (indexed by a genetic variant) could also enhance biological understanding and provide insights relevant to the identification and prioritization of potential therapeutic targets, such as the re-purposing of existing therapies to new disease indications and the anticipation of safety and efficacy signals in clinical trials. One notable example has been our demonstration, following a phenome scan across a wide range of traits and diseases, that genetic variants that upregulate the interleukin-1 receptor antagonist are associated with a higher risk of coronary artery disease, partly mediated through elevation of pro-atherogenic lipids (Interleukin 1 Genetics Consortium *et al.*, 2015). So far, however, it has been difficult to generalize this approach, partly because the collation of associations with many phenotypes can be time-consuming, especially if information is sought about multiple variants.

Catalogues of GWAS results already exist, such as the NHGRI-EBI GWAS catalog (Welter *et al.*, 2014), as well as data repositories, e.g. dbGaP (Mailman *et al.*, 2007). However, these either focus on variants robustly associated with a particular trait (and hence do not take advantage of the wide range of publicly available full GWAS results), do not contain estimates or directions of effect, and/or are difficult to search in a systematic way. Also, the results often have inconsistent formats and the output for each variant is not necessarily given according to the same effect allele. In addition, most catalogues of GWAS do not identify associations with proxy variants, which means that if an association between the variant of interest and a trait is unavailable, a suitable proxy must be found using a separate resource and then searched in the catalogue. Some of the latest variant annotation tools (which include proxy look-ups) do contain results from the NHGRI-EBI GWAS catalog (e.g. SNiPA; Arnold *et al.*, 2014), however, they only return *P* values. To help address these issues, we developed a web-based tool ‘PhenoScanner’ that extracts and aligns associations for user-specified variants and proxies across a large curated database.

## 2 Methods

PhenoScanner consists of a Perl interface (with R command line tool) that connects to a MySQL database. To develop the initial database, we collated 137 genotype–phenotype association datasets, including results for anthropometric traits, blood pressure, lipids, cardiometabolic diseases, renal function measures, glycemic traits, inflammatory diseases, psychiatric diseases and smoking phenotypes (Supplementary Table). We also included the NHGRI-EBI GWAS catalog, NHLBI GRASP (Leslie *et al.*, 2014) and dbGaP catalogues of associations. To ensure consistent formatting, we aligned alleles to the plus strand, added or updated chromosome positions to build 37 using dbSNP (release 138) (Sherry *et al.*, 2001) and liftOver (https://genome.ucsc.edu/cgi-bin/hgLiftOver), and updated old rsIDs to dbSNP release 141 (Supplementary Data). Linkage disequilibrium (LD) measures between neighbouring variants in the autosomal chromosomes were calculated using the phased haplotypes from European samples in 1000 Genomes phase 3 (*N* = 503) (1000 Genomes Project Consortium *et al.*, 2012). Variants with minor allele frequencies <0.5% were removed along with multiallelic variants and large indels (≥5 bases). For each remaining variant, we calculated D′ and *r*^2^ for variants within 500 kb in either direction, and kept LD statistics for pairs of variants with $r^{2}\geq 0.6$. LD statistics based on the CEU population from Hapmap 2 release 24 (Frazer *et al.*, 2007) are also available (Supplementary Data).

The user may enter either one variant into the text box on the website or upload up to 50 variants in a text file. The Perl interface annotates the variant alleles using dbSNP, identifies proxies of the specified variants (if requested) in the database according to a user-specified pairwise *r*^2^ threshold, and queries the catalogue of genotype–phenotype associations for the specified variants and their proxies. Association results are collated and presented with respect to the same effect and non-effect alleles for each variant. The associations with proxies are aligned according to the effect and non-effect alleles of the corresponding primary variant of interest for added ease of interpretation. The output is a file of associations, which is made available to download. There is also a *P* value filter option that only retains results with study-specific *P* values less than the selected threshold.

## 3 Results

**Fig. 1.**
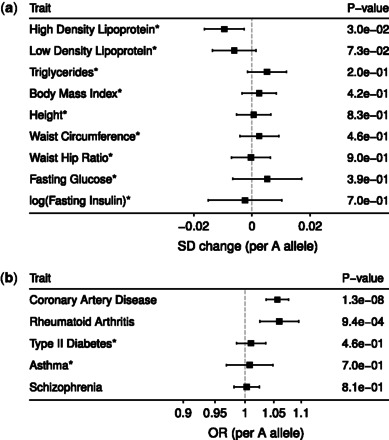
Association results for rs10840293 with a subset of the traits (a) and diseases (b) available in PhenoScanner. An asterisk indicates the use of a proxy variant (rs93138; $r^{2}=0.95$) in reporting the association. SD, standard deviation; OR, odds ratio

To illustrate the use of PhenoScanner, we ran the program with rs10840293 (an intronic variant in *SWAP70*) using proxies from 1000 Genomes and a *r*^2^ cut-off of 0.8. The program found and aligned over 1000 associations with either rs10840293 or a proxy of rs10840293 ($r^{2}\geq 0.8$) in <10s (Fig. 1 and Supplementary Data). Hence, even though associations between rs10840293 and phenotypes are mostly unavailable, we were able to obtain a range of related associations using proxies (e.g. rs93138 in Fig. 1).

## 4 Conclusion

In summary, PhenoScanner is a large curated database of publicly available summary results from genetic association studies. This database extends current catalogues of genetic data by including all available results as opposed to filtering on strength of association. Moreover, PhenoScanner aligns genotype–phenotype associations across traits and proxies, providing the user with an easily interpretable formatted output file. We anticipate that this tool will make cross-referencing genetic variants with many phenotypes faster and more efficient.

## Funding

This work was supported by the UK Medical Research Council [G66840, G0800270], Pfizer [G73632], British Heart Foundation [SP/09/002], UK National Institute for Health Research Cambridge Biomedical Research Centre, European Research Council [268834], and European Commission Framework Programme 7 [HEALTH-F2-2012-279233]. 

*Conflict of Interest*: none declared.

## Supplementary Material

Supplementary Data
