# Supplementary material for: PhenoScanner: a database of human genotype–phenotype associations
Source: Bioinformatics. 2016 Jun 17;32(20):3207–9. doi: 10.1093/bioinformatics/btw373 (PMC5048068; doi:10.1093/bioinformatics/btw373)
Supplement: Supplementary Data [file btw373_supplementary_data.zip › suppl_data.zip!/PhenoScanner_Supplementary.pdf]

PhenoScanner: a database of human genotype-phenotype associations

Supplementary Data

James R Staley[1,*], James Blackshaw[1], Mihir A Kamat[1], Steve Ellis[1], Praveen Surendran[1], Benjamin B Sun[1], Dirk S Paul[1], Daniel Freitag[1], Stephen Burgess[1], John Danesh[1,2,3], Robin Young[1,4,†], Adam S Butterworth[1,3,†].

[1]Cardiovascular Epidemiology Unit, Department of Public Health and Primary Care, University of Cambridge, Cambridge, CB1 8RN, United Kingdom, [2] Wellcome Trust Sanger Institute, Hinxton, CB10 1SA, United Kingdom, [3] NIHR Blood and Transplant Research Unit, Department of Public Health and Primary Care, University of Cambridge, Cambridge, CB1 8RN, United Kingdom, [4]Robertson Centre for Biostatistics, University of Glasgow, Glasgow, G12 8QQ, United Kingdom.

[*] To whom correspondence should be addressed.

[†] Jointly supervised the work.

Development of the catalogue of genotype-phenotype associations

We developed the initial database of genotype-phenotype associations by collating summary results for anthropometric traits, blood pressure, lipids, cardiometabolic diseases, renal function measures, glycemic traits, early growth traits, bowel disease, bone-density, pancreatic cancer, asthma, psychiatric diseases, smoking phenotypes, adiponectin, leptin, breast cancer, body fat percentage, heart rate, prostate cancer, statin response, visual refractive error, valvular calcification, aortic stenosis, age macular degeneration, and epilepsy (Supplementary Table). These datasets were obtained from the GIANT,[1–7] ICBP,[8] GLGC,[9,10] CARDIoGRAM,[11] C4D,[12] CARDIoGRAMplusC4D,[13,14] DIAGRAM,[15,16] Okada Y et al,[17] CKD-Gen,[18,19] MAGIC,[20–25] EGGC,[26–28] IBDGC,[29] GEFOS,[30] PanScan,[31] GABRIEL,[32] PGC,[33–37] CONVERGE,[38] TAG,[39] ADIPOGen,[40] Kilpelainen T et al,[41] Lu Y et al,[42] HRGene,[43] Hunter D et al,[44] Yeager M et al,[45] Barber M et al,[46] Stambolian D et al,[47] CHARGE,[48] AMD Gene,[49] and ILAE[50] consortia. In addition to the full GWAS summary results datasets, we also included the NHGRI-EBI GWAS catalog,[51] NHLBI GRASP[52] and dbGaP[53] catalogues of associations. Links to the websites where these datasets were downloaded are provided below. The following variables were retained (if available) from each dataset or obtained from the relevant publication: phenotype name, consortia name or lead author, pubmed ID, year of publication, rsID, chromosome, position (build 37), effect allele, non-effect allele, effect allele frequency, minor allele frequency, effect estimate, standard error, p-value, direction of effect, unit of association, number of individuals, number of (disease) cases, number of controls, number of studies, and p-value of heterogeneity across studies. If build 37 chromosome positions were unavailable, we either added them using dbSNP (release 138)[54] or updated build 36 chromosome positions using liftover (https://genome.ucsc.edu/cgi-bin/hgLiftOver). rsIDs were updated to those used in dbSNP release 141. The datasets with reported alleles were checked to see if the results were aligned to the (build 37) plus strand. All the datasets except the NHGRI-EBI GWAS catalog had >98% of results aligned to the plus strand. The results from the NHGRI-EBI GWAS catalog for variants that are 'not palindromic' (not A/T or G/C) were aligned to the plus strand and palindromic variants were checked against the corresponding reference; only effect estimates and standard errors aligned to the plus strand were retained. Measures of disease risk (e.g. odds ratios ratios/hazard ratios) were put on the log-scale and standard errors were computed using either confidence intervals or p-values if unavailable.

Links to websites where the datasets were downloaded

GIANT - anthropometric measures:

http://www.broadinstitute.org/collaboration/giant/index.php/GIANT_consortium_data_files

ICBP - systolic and diastolic blood pressure:

See supplementary materials provided with the publication[8]

GLGC - lipids and triglycerides:

http://csg.sph.umich.edu/abecasis/public

CARDIoGRAM, C4D, CARDIoGRAMplusC4D - coronary artery disease:

http://www.cardiogramplusc4d.org/downloads

DIAGRAM - type II diabetes:

http://diagram-consortium.org/downloads.html

Okada Y et al - rheumatoid arthritis:

http://plaza.umin.ac.jp/~yokada/datasource/software.htm

CKD-Gen - renal function measures:

http://www.nhlbi.nih.gov/research/intramural/researchers/pi/fox-caroline/datasets

MAGIC - glycemic traits:

http://www.magicinvestigators.org/downloads

EGGC - early growth traits:

http://egg-consortium.org

IBDGC - bowel disease:

http://www.ibdgenetics.org/downloads.html

GEFOS - bone density (see list of GEFOS collaborators at the end of supplementary):

http://www.gefos.org/?q=content/data-release-2012

PanScan - pancreatic cancer:

http://www.ncbi.nlm.nih.gov/gap/?term=PanScan

PGC - psychiatric diseases:

https://www.med.unc.edu/pgc/downloads

CONVERGE - major depression disorder:

https://www.med.unc.edu/pgc/downloads

TAG - smoking phenotypes:

https://www.med.unc.edu/pgc/downloads

GABRIEL - asthma:

http://www.cng.fr/gabriel/results.html

ADIPOGen - adiponectin:

http://www.mcgill.ca/genepi/adipogen-consortium

Kilpelainen T et al - leptin:

https://walker05.u.hpc.mssm.edu/

Lu Y et al - body fat percentage:

https://walker05.u.hpc.mssm.edu/

HRGene - heart rate:

https://walker05.u.hpc.mssm.edu/

Hunter D et al - breast cancer:

ftp://ftp.ncbi.nlm.nih.gov/dbgap/studies/phs000147/analyses/

Yeager M et al - prostate cancer:

ftp://ftp.ncbi.nlm.nih.gov/dbgap/studies/phs000207/analyses/

Barber M et al - response to statins:

http://stephenslab.uchicago.edu/BarberKrauss2010SuppData.html

Stambolian D et al - visual refractive errors:

http://hmg.oxfordjournals.org/content/suppl/2013/03/11/ddt116.DC1

CHARGE - valvular calcification and aortic stenosis:

https://www.framinghamheartstudy.org/researchers/gwas-study-results/index.php

AMD Gene - age macular degeneration:

http://www.sph.umich.edu/csg/abecasis/public/amdgene2012/

ILAE - epilepsy:

http://www.epigad.org

NHGRI-EBI GWAS catalog:

http://www.ebi.ac.uk/gwas/docs/downloads

NHLBI GRASP:

http://grasp.nhlbi.nih.gov/Overview.aspx

dbGaP:

http://www.ncbi.nlm.nih.gov/projects/gapplusprev/sgap_plus.htm

Linkage disequilibrium statistics

1000 Genomes phase 3

The linkage disequilibrium (LD) statistics from the European samples in 1000 Genomes phase 3[55] were computed using Tabix[56] and R (version 3.0.2).

Hapmap 2

LD statistics from Hapmap 2[57] were computed using the genotypes from the independent individuals from the CEU population (N=60). These LD statistics were calculated with Plink 1.9 (https://www.cog-genomics.org/plink2)[58] using the dprime option, which computes Lewontin's D-prime statistic as well as basing the LD statistics on the maximum likelihood solution to the cubic equation.[59] For each variant, we calculated D' and r^2 for variants within 500kb in either direction, and kept LD statistics for pairs of variants with $r^2 \geq 0.6$.

Example

The full results and proxy information of rs10840293 are available in the accompanying CSV files. Data used in the example were obtained from GLGC,[10] GIANT,[4–6] MAGIC,[21] CARDIoGRAMplusC4D,[14] Okada et al,[17] DIAGRAM[15] and GABRIEL[32] consortia. We transformed the results for fasting glucose and fasting insulin to ensure that all results are on the standard deviation scale (standard deviations used were 0.63 mmol/l for fasting glucose and 0.62 log-pmol/l for fasting insulin). The lipid and adiposity measures were inverse normally rank transformed.

Batch upload

The maximum number of input variants in the batch upload is 50. If the request to the genotype-phenotype association database is going be over 500 genetic variants including proxies, to ensure fast look-ups, the input variants with more than 10 proxies will be restricted to the 10 proxies (including the input variant itself) with the highest r^2 for the genotype-phenotype association look-ups.

Acknowledgements

We would like to thank Andrew Johnson (NHLBI) for allowing us to host the NHLBI GRASP catalogue, the PGC and TAG management groups for allowing us to host their results, and all the other data providers.

References

1. Yang J, Loos RJ, Powell JE, Medland SE, Speliotes EK, Chasman DI, et al. FTO genotype is associated with phenotypic variability of body mass index. *Nature*. 2012;490(7419):267–272.

2. Berndt SI, Gustafsson S, Mägi R, Ganna A, Wheeler E, Feitosa MF, et al. Genome-wide meta-analysis identifies 11 new loci for anthropometric traits and provides insights into genetic architecture. *Nat Genet*. 2013;45(5):501–512.

3. Randall JC, Winkler TW, Kutalik Z, Berndt SI, Jackson AU, Monda KL, et al. Sex-stratified genome-wide association studies including 270,000 individuals show sexual dimorphism in genetic loci for anthropometric traits. *PLoS Genet*. 2013;9(6):e1003500.

4. Wood AR, Esko T, Yang J, Vedantam S, Pers TH, Gustafsson S, et al. Defining the role of common variation in the genomic and biological architecture of adult human height. *Nat Genet*. 2014;46(11):1173–1186.

5. Shungin D, Winkler TW, Croteau-Chonka DC, Ferreira T, Locke AE, Mägi R, et al. New genetic loci link adipose and insulin biology to body fat distribution. *Nature*. 2015;518(7538):187–196.

6. Locke AE, Kahali B, Berndt SI, Justice AE, Pers TH, Day FR, et al. Genetic studies of body mass index yield new insights for obesity biology. *Nature*. 2015;518(7538):197–206.

7. Winkler TW, Justice AE, Graff M, Barata L, Feitosa MF, Chu S, et al. The influence of age and sex on genetic associations with adult body size and shape: a large-scale genome-wide interaction study. *PLoS Genet*. 2015;11(10):e1005378.

8. International Consortium for Blood Pressure Genome-Wide Association Studies, et al. Genetic variants in novel pathways influence blood pressure and cardiovascular disease risk. *Nature*. 2011;478(7367):103–109.

9. Teslovich TM, Musunuru K, Smith AV, Edmondson AC, Stylianou IM, Koseki M, et al. Biological, clinical and population relevance of 95 loci for blood lipids. *Nature.* 2010;466(7307):707–713.

10. Global Lipids Genetics Consortium, et al. Discovery and refinement of loci associated with lipid levels. *Nat Genet.* 2013;45(11):1274–1283.

11. Schunkert H, König IR, Kathiresan S, Reilly MP, Assimes TL, Holm H, et al. Large-scale association analysis identifies 13 new susceptibility loci for coronary artery disease. *Nat Genet.* 2011;43(4):333–338.

12. Coronary Artery Disease (C4D) Genetics Consortium, et al. A genome-wide association study in Europeans and South Asians identifies five new loci for coronary artery disease. *Nat Genet.* 2011;43(4):339–344.

13. Deloukas P, Kanoni S, Willenborg C, Farrall M, Assimes TL, Thompson JR, et al. Large-scale association analysis identifies new risk loci for coronary artery disease. *Nat Genet.* 2013;45(1):25–33.

14. CARDIoGRAMplusC4D Consortium, et al. A comprehensive 1000 Genomes-based genome-wide association meta-analysis of coronary artery disease. *Nat Genet.* 2015;47(10):1121–1130.

15. DIAbetes Genetics Replication And Meta-analysis (DIAGRAM) Consortium, et al. Genome-wide trans-ancestry meta-analysis provides insight into the genetic architecture of type 2 diabetes susceptibility. *Nat Genet.* 2014;46(3):234–244.

16. Gaulton KJ, Ferreira T, Lee Y, Raimondo A, Mägi R, Reschen ME, et al. Genetic fine mapping and genomic annotation defines causal mechanisms at type 2 diabetes susceptibility loci. *Nat Genet.* 2015;47(12):1415–1425.

17. Okada Y, Wu D, Trynka G, Raj T, Terao C, Ikari K, et al. Genetics of rheumatoid arthritis contributes to biology and drug discovery. *Nature.* 2014;506(7488):376–381.

18. Köttgen A, Pattaro C, Böger C, Fuchsberger C, Olden M, Glazer NL, et al. New loci associated with kidney function and chronic kidney disease. *Nat Genet.* 2010;42(5):376–384.

19. Böger CA, Chen MH, Tin A, Olden M, Köttgen A, De Boer IH, et al. CUBN is a gene locus for albuminuria. *JASN.* 2011;22(3):555–570.

20. Saxena R, Hivert MF, Langenberg C, Tanaka T, Pankow JS, Vollenweider P, et al. Genetic variation in GIPR influences the glucose and insulin responses to an oral glucose challenge. *Nat Genet.* 2010;42(2):142–148.

21. Dupuis J, Langenberg C, Prokopenko I, Saxena R, Soranzo N, Jackson AU, et al. New genetic loci implicated in fasting glucose homeostasis and their impact on type 2 diabetes risk. *Nat Genet.* 2010;42(2):105–116.

22. Soranzo N, Sanna S, Wheeler E, Gieger C, Radke D, Dupuis J, et al. Common variants at 10 genomic loci influence hemoglobin A1C levels via glycemic and nonglycemic pathways. *Diabetes.* 2010;59(12):3229–3239.

23. Strawbridge RJ, Dupuis J, Prokopenko I, Barker A, Ahlqvist E, Rybin D, et al. Genome-wide association identifies nine common variants associated with fasting proinsulin levels and provides new insights into the pathophysiology of type 2 diabetes. *Diabetes.* 2011;60(10):2624–2634.

24. Scott RA, Lagou V, Welch RP, Wheeler E, Montasser ME, Luan J, et al. Large-scale association analyses identify new loci influencing glycemic traits and provide insight into the underlying biological pathways. *Nat Genet.* 2012;44(9):991–1005.

25. Prokopenko I, Poon W, Mägi R, Prasad B, Salehi SA, Almgren P, et al. A central role for GRB10 in regulation of islet function in man. *PLoS Genet.* 2014;10(4):e1004235.

26. Early Growth Genetics (EGG) Consortium, et al. A genome-wide association meta-analysis identifies new childhood obesity loci. *Nat Genet.* 2012;44(5):526–531.

27. Taal HR, St Pourcain B, Thiering E, Das S, Mook-Kanamori DO, Warrington NM, et al. Common variants at 12q15 and 12q24 are associated with infant head circumference. *Nat Genet.* 2012;44(5):532–538.

28. Horikoshi M, Yaghootkar H, Mook-Kanamori DO, Sovio U, Taal HR, Hennig BJ, et al. New loci associated with birth weight identify genetic links between intrauterine growth and adult height and metabolism. *Nat Genet.* 2013;45(1):76–82.

29. Jostins L, Ripke S, Weersma RK, Duerr RH, McGovern DP, Hui KY, et al. Host-microbe interactions have shaped the genetic architecture of inflammatory bowel disease. *Nature.* 2012;491(7422):119–124.

30. Estrada K, Styrkarsdottir U, Evangelou E, Hsu YH, Duncan EL, Ntzani EE, et al. Genome-wide meta-analysis identifies 56 bone mineral density loci and reveals 14 loci associated with risk of fracture. *Nat Genet.* 2012;44(5):491–501.

31. Petersen GM, Amundadottir L, Fuchs CS, Kraft P, Stolzenberg-Solomon RZ, Jacobs KB, et al. A genome-wide association study identifies pancreatic cancer susceptibility loci on chromosomes 13q22. 1, 1q32. 1 and 5p15. 33. *Nat Genet.* 2010;42(3):224–228.

32. Moffatt MF, Gut IG, Demenais F, Strachan DP, Bouzigon E, Heath S, et al. A large-scale, consortium-based genomewide association study of asthma. *N Eng J Med.* 2010;363(13):1211–1221.

33. Neale BM, Medland SE, Ripke S, Asherson P, Franke B, Lesch KP, et al. Meta-analysis of genome-wide association studies of attention-deficit/hyperactivity disorder. *J Am Acad Child Adolesc Psychiatry.* 2010;49(9):884–897.

34. Sklar P, Ripke S, Scott LJ, Andreassen OA, Cichon S, Craddock N, et al. Large-scale genome-wide association analysis of bipolar disorder identifies a new susceptibility locus near ODZ4. *Nat Genet.* 2011;43(10):977.

35. Cross-Disorder Group of the Psychiatric Genomics Consortium, et al. Identification of risk loci with shared effects on five major psychiatric disorders: a genome-wide analysis. *Lancet.* 2013;381(9875):1371–1379.

36. Ripke S, Wray NR, Lewis CM, Hamilton SP, Weissman MM, Breen G, et al. A mega-analysis of genome-wide association studies for major depressive disorder. *Mol Psychiatry.* 2013;18(4):497–511.

37. Schizophrenia Working Group of the Psychiatric Genomics Consortium, et al. Biological insights from 108 schizophrenia-associated genetic loci. *Nature.* 2014;511(7510):421–427.

38. Cai N, Bigdeli TB, Kretzschmar W, Li Y, Liang J, Song L, et al. Sparse whole-genome sequencing identifies two loci for major depressive disorder. *Nature.* 2015;523:588–591.

39. Tobacco and Genetics Consortium, et al. Genome-wide meta-analyses identify multiple loci associated with smoking behavior. *Nat Genet.* 2010;42:441–447.

40. Dastani Z, Hivert MF, Timpson N, Perry JR, Yuan X, Scott RA, et al. Novel loci for adiponectin levels and their influence on type 2 diabetes and metabolic traits: a multi-ethnic meta-analysis of 45,891 individuals. *PLoS Genet*. 2012;8(3):e1002607.

41. Kilpeläinen TO, Carli JFM, Skowronski AA, Sun Q, Kriebel J, Feitosa MF, et al. Genome-wide meta-analysis uncovers novel loci influencing circulating leptin levels. *Nat Commun*. 2016;7.

42. Lu Y, Bishop D, et al. New loci for body fat percentage reveal link between adiposity and cardiometabolic disease risk. *Nat Commun*. 2015;.

43. den Hoed M, Eijgelsheim M, Esko T, Brundel BJ, Peal DS, Evans DM, et al. Identification of heart rate-associated loci and their effects on cardiac conduction and rhythm disorders. *Nat Genet*. 2013;45(6):621–631.

44. Hunter DJ, Kraft P, Jacobs KB, Cox DG, Yeager M, Hankinson SE, et al. A genome-wide association study identifies alleles in FGFR2 associated with risk of sporadic postmenopausal breast cancer. *Nat Genet*. 2007;39(7):870–874.

45. Yeager M, Orr N, Hayes RB, Jacobs KB, Kraft P, Wacholder S, et al. Genome-wide association study of prostate cancer identifies a second risk locus at 8q24. *Nat Genet*. 2007;39(5):645–649.

46. Barber MJ, Mangravite LM, Hyde CL, Chasman DI, Smith JD, McCarty CA, et al. Genome-wide association of lipid-lowering response to statins in combined study populations. *PloS one*. 2010;5(3):e9763.

47. Stambolian D, Wojciechowski R, Oexle K, Pirastu M, Li X, Raffel LJ, et al. Meta-analysis of genome-wide association studies in five cohorts reveals common variants in RBFOX1, a regulator of tissue-specific splicing, associated with refractive error. *Human molecular genetics*. 2013;p. ddt116.

48. Thanassoulis G, Campbell CY, Owens DS, Smith JG, Smith AV, Peloso GM, et al. Genetic associations with valvular calcification and aortic stenosis. *N Eng J Med*. 2013;368(6):503–512.

49. AMD Gene Consortium, et al. Seven new loci associated with age-related macular degeneration. *Nat Genet*. 2013;45(4):433–439.

50. The International League Against Epilepsy Consortium, et al. Genetic determinants of common epilepsies: a meta-analysis of genome-wide association studies. *Lancet Neurol*. 2014;13(9):893.

51. Welter D, MacArthur J, Morales J, Burdett T, Hall P, Junkins H, et al. The NHGRI GWAS Catalog, a curated resource of SNP-trait associations. *Nucleic Acids Res.* 2014;42(D1):D1001–D1006.

52. Leslie R, O'Donnell CJ, Johnson AD. GRASP: analysis of genotype–phenotype results from 1390 genome-wide association studies and corresponding open access database. *Bioinformatics.* 2014;30(12):i185–i194.

53. Mailman MD, Feolo M, Jin Y, Kimura M, Tryka K, Bagoutdinov R, et al. The NCBI dbGaP database of genotypes and phenotypes. *Nat Genet.* 2007;39(10):1181–1186.

54. Sherry ST, Ward MH, Kholodov M, Baker J, Phan L, Smigielski EM, et al. dbSNP: the NCBI database of genetic variation. *Nucleic Acids Res.* 2001;29(1):308–311.

55. 1000 Genomes Project Consortium, et al. An integrated map of genetic variation from 1,092 human genomes. *Nature.* 2012;491(7422):56–65.

56. Li H. Tabix: fast retrieval of sequence features from generic TAB-delimited files. *Bioinformatics.* 2011;27(5):718–719.

57. Frazer KA, Ballinger DG, Cox DR, Hinds DA, Stuve LL, Gibbs RA, et al. A second generation human haplotype map of over 3.1 million SNPs. *Nature.* 2007;449(7164):851–861.

58. Chang CC, Chow CC, Tellier L, Vattikuti S, Purcell SM, Lee JJ. Second-generation PLINK: rising to the challenge of larger and richer datasets. *Gigascience.* 2015;4(7).

59. Gaunt TR, Rodríguez S, Day IN. Cubic exact solutions for the estimation of pairwise haplotype frequencies: implications for linkage disequilibrium analyses and a web tool 'CubeX'. *BMC Bioinformatics.* 2007;8(1):428.

Supplementary Table: Genetic association studies available in the first release of PhenoScanner

Study	Phenotype(s)	PMID	N	N SNPs
Genotype-phenotype Association Datasets				
ADIPOGen	Adiponectin	22479202	29,347	2,675,208
AMD Gene	Age Magular Degeneration (All, Geographic Atrophy, Neovascularization)	23455636	59,494	2,422,876
Barber M et al.	Response to Statin Treatment (HDL, LDL, Triglycerides, Total Cholesterol)	20339536	3,932	2,588,920
C4D	Coronary Artery Disease	21378988	30,482	540,232
CARDIoGRAM	Coronary Artery Disease	21378990	86,995	2,420,360
CARDIoGRAMplusC4D	Coronary Artery Disease	23202125	194,427	79,128
CARDIoGRAMplusC4D	Coronary Artery Disease	26343387	184,305	9,455,778
CHARGE	Aortic Valve Calcification, Mitral Annular Calcification	23388002	6,942	2,499,695
CKD-Gen	eGFR (based on creatinine or cystatin C), CKD	20383146	67,093	2,769,999
CKD-Gen	Microalbuminuria, Urinary Albumin-Creatinine Ratio	21355061	31,580	2,758,870
CONVERGE	Major Depressive Disorder	26176920	10,640	6,208,599
DIAGRAM	Type II Diabetes	24509480	110,452	2,915,011
DIAGRAM	Type II Diabetes	26551672	84,780	45,943
EGGC	Birth Weight	23202124	26,836	2,684,393
EGGC	Infant Head Circumference	22504419	10,768	2,449,806
EGGC	Childhood Obesity	22484627	13,848	2,442,738
GABRIEL	Asthma	20860503	26,475	567,589
GEFOS	Lumbar Spine Bone Mineral Density, Femoral Neck Bone Mineral Density	22504420	32,961	2,478,338
GIANT	Body Mass Index Variability, Height Variability	22982992	129,201	2,417,848
GIANT	Body Mass Index, Height and Waist Hip Ratio Tails, Obesity Classes 1, 2 and 3, Overweight	23563607	158,855	1,984,813
GIANT	Height, Weight, Body Mass Index, Waist Circumference, Waist Hip Ratio	23754948	75,819	2,762,771
GIANT	Height	25282103	253,280	2,550,858
GIANT	Waist Circumference, Waist Hip Ratio, Hip	25673412	244,441	2,566,629
GIANT	Body Mass Index	25673413	339,224	2,555,510
GIANT	Age and Sex straitified Body Mass Index and Waist Hip Ratio	26426971	73,558	2,407,663
GLGC	HDL, LDL, Total Cholesterol, Triglycerides	20686565	100,184	2,692,564
GLGC	HDL, LDL, Total Cholesterol, Triglycerides	24097068	188,577	2,437,751
HRGene	Heart Rate	23583979	85,787	2,488,384
Hunter D et al.	Breast Cancer	17529973	2,287	471,011
IBDGC	Crohn's Disease, Ulcerative Colitis	23128233	20,672	1,428,749
ICBP	Systolic Blood Pressure, Diastolic Blood Pressure	21909115	69,395	2,461,325
ILAE	Epilepsy, Focal Epilepsy, Genetic Generalised Epilepsy	25087078	34,853	5,968,968
Kilpelainen T et al.	Leptin (plus adjusted for Body Mass Index)	26833098	32,161	2,473,865
Lu Y et al.	Body Fat Percentage	26833246	100,716	3,228,052
MAGIC	2hr Fasting Glucose	20081857	15,234	2,401,708
MAGIC	Fasting Glucose, Fasting Insulin, HOMA-B, HOMA-IR	20081858	46,186	2,470,476
MAGIC	HbA1c	20858683	46,368	2,572,402
MAGIC	Proinsulin	21873549	10,701	2,496,073
MAGIC	2hr Fasting Glucose, Fasting Glucose, Fasting Insulin (plus adjusted for Body Mass Index)	22885924	133,010	64,435
MAGIC	Insulin secretion during OGTT (9 GSIS indices)	24699409	5,318	2,433,996
Okada Y et al.	Rheumatoid Arthritis (Europeans, Asians, Trans-Ethnic)	24390342	80,799	9,739,303
PanScan	Pancreatic Cancer	20101243	7,785	543,436
PGC	Attention Deficit Hyperactivity Disorder	20732625	3,351	1,206,461
PGC	Bipolar Disorder	21926972	16,731	2,427,220
PGC	Cross Disorder	23453885	61,220	1,249,699
PGC	Major Depressive Disorder	22472876	18,759	1,235,109
PGC	Schizophrenia	25056061	32,295	9,444,230
Stambolian D et al.	Visual Refractive Error	23474815	7,280	2,574,241
TAG	Ever Smoker, Former Smoker, Age of Onset of Smoking, Cigarettes per Day	20418890	74,035	2,459,118
Yeager M et al.	Prostate Cancer	17401363	2,307	473,737
GWAS Catalogues				
dbGaP	Various	17898773	Various	28,766
NHGRI-EBI GWAS Catalog	Various	24316577	Various	11,912
NHLBI GRASP (version 2)	Various	25428361	Various	8,870,000

PMID, PubMed ID; HDL, high density lipoprotein; LDL, low density lipoprotein; eGFR, estimated glomerular filtration rate; CKD, chronic kidney disease.

GEFOS collaborators

Karol Estrada[1,2,3], Unnur Styrkarsdottir[4,139], Evangelos Evangelou[5,139], Yi-Hsiang Hsu[6,7,139], Emma L Duncan[8,9,139], Evangelia E Ntzani[5,139], Ling Oei[1,2,3,139], Omar M E Albagha[10], Najaf Amin[2], John P Kemp[11], Daniel L Koller[12], Guo Li[13], Ching-Ti Liu[14], Ryan L Minster[15], Alireza Moayyeri[16,17], Liesbeth Vandenput[18], Dana Willner[8,19], Su-Mei Xiao[20,21], Laura M Yerges-Armstrong[22], Hou-Feng Zheng[23], Nerea Alonso[10], Joel Eriksson[18], Candace M Kammerer[15], Stephen K Kaptoge[16], Paul J Leo[8], Gudmar Thorleifsson[4], Scott G Wilson[17,24,25], James F Wilson[26,27], Ville Aalto[28,29], Markku Alen[30], Aaron K Aragaki[31], Thor Aspelund[32,33], Jacqueline R Center[34,35,36], Zoe Dailiana[37], David J Duggan[38], Melissa Garcia[39], Natàlia Garcia-Giralt[40], Sylvie Giroux[41], Göran Hallmans[42], Lynne J Hocking[43], Lise Bjerre Husted[44], Karen A Jameson[45], Rita Khusainova[46,47], Ghi Su Kim[48], Charles Kooperberg[31], Theodora Koromila[49], Marcin Kruk[50], Marika Laaksonen[51], Andrea Z Lacroix[31], Seung Hun Lee[48], Ping C Leung[52], Joshua R Lewis[24,25], Laura Masi[53], Simona Mencej-Bedrac[54], Tuan V Nguyen[34,35], Xavier Nogues[40], Millan S Patel[55], Janez Prezelj[56], Lynda M Rose[57], Serena Scollen[58], Kristin Siggeirsdottir[32], Albert V Smith[32,33], Olle Svensson[59], Stella Trompet[60,61], Olivia Trummer[62], Natasja M van Schoor[63], Jean Woo[64], Kun Zhu[24,25], Susana Balcells[65], Maria Luisa Brandi[53], Brendan M Buckley[66], Sulin Cheng[67,68], Claus Christiansen[69], Cyrus Cooper[45], George Dedoussis[70], Ian Ford[71], Morten Frost[72,73], David Goltzman[74], Jesús González-Macías[75,76], Mika Kähönen[77,78], Magnus Karlsson[79], Elza Khusnutdinova[46,47], Jung-Min Koh[48], Panagoula Kollia[49], Bente Lomholt Langdahl[44], William D Leslie[80], Paul Lips[81,82], Östen Ljunggren[83], Roman S Lorenc[50], Janja Marc[54], Dan Mellström[18], Barbara Obermayer-Pietsch[62], José M Olmos[75,76], Ulrika Pettersson-Kymmer[84], David M Reid[43], José A Riancho[75,76], Paul M Ridker[57,85], François Rousseau[41,86,87], P Eline Slagboom[88,3], Nelson LS Tang[89,90], Roser Urreizti[65], Wim Van Hul[91], Jorma Viikari[92,93], María T Zarrabeitia[94], Yurii S Aulchenko[2], Martha Castano-Betancourt[1,2,3], Elin Grundberg[95,96,97], Lizbeth Herrera[1], Thorvaldur Ingvarsson[98,99,33], Hrefna Johannsdottir[4], Tony Kwan[95,96], Rui Li[100], Robert Luben[16], Carolina Medina-Gómez[1,2], Stefan Th Palsson[4], Sjur Reppe[101], Jerome I Rotter[102], Gunnar Sigurdsson[103,33], Joyce B J van Meurs[1,2,3], Dominique Verlaan[95,96], Frances MK Williams[17], Andrew R Wood[104], Yanhua Zhou[14], Kaare M Gautvik[101,105,106], Tomi Pastinen[95,96,107], Soumya Raychaudhuri[108,109], Jane A Cauley[110], Daniel I Chasman[57,85], Graeme R Clark[8], Steven R Cummings[111], Patrick Danoy[8], Elaine M Dennison[45], Richard Eastell[112], John A Eisman[34,35,36], Vilmundur Gudnason[32,33], Albert Hofman[2,3], Rebecca D Jackson[113,114], Graeme Jones[115], J Wouter Jukema[60,116,117], Kay-Tee Khaw[16], Terho Lehtimäki[118,119], Yongmei Liu[120], Mattias Lorentzon[18], Eugene McCloskey[112,121], Braxton D Mitchell[22], Kannabiran Nandakumar[6,7], Geoffrey C Nicholson[122], Ben A Oostra[123], Munro Peacock[124], Huibert A P Pols[1,2], Richard L Prince[24,25], Olli Raitakari[28,29], Ian R Reid[125], John Robbins[126], Philip N Sambrook[127], Pak Chung Sham[128,129], Alan R Shuldiner[22,130], Frances A Tylavsky[131], Cornelia M van Duijn[2], Nick J Wareham[132], L Adrienne Cupples[14,133], Michael J Econs[124,12], David M Evans[11], Tamara B Harris[39], Annie Wai Chee Kung[20,21], Bruce M Psaty[134,135], Jonathan Reeve[136], Timothy D Spector[17], Elizabeth A Streeten[22,130], M Carola Zillikens[1], Unnur Thorsteinsdottir[4,33,140], Claes Ohlsson[18,140], David Karasik[6,7,140], J Brent Richards[137,17,140], Matthew A Brown[8,140], Kari Stefansson[4,33,140], André G Uitterlinden[1,2,3,140], Stuart H Ralston[10,140], John P A Ioannidis[138,5,140], Douglas P Kiel[6,7,140], Fernando Rivadeneira[1,2,3,140].

Affiliations:

[1]Department of Internal Medicine, Erasmus Medical Center, Rotterdam, The Netherlands. [2]Department of Epidemiology,

Erasmus Medical Center, Rotterdam, The Netherlands. [3]Netherlands Genomics Initiative (NGI)-sponsored Netherlands Consortium for Healthy Aging (NCHA), Leiden, The Netherlands. [4]deCODE Genetics, Reykjavik, Iceland. [5]Department of Hygiene and Epidemiology, University of Ioannina, Ioannina, Greece. [6]Institute for Aging Research, Hebrew SeniorLife, Boston, USA. [7]Department of Medicine, Harvard Medical School, Boston, USA. [8]Human Genetics Group, University of Queensland Diamantina Institute, Brisbane, Australia. [9]Department of Endocrinology, Royal Brisbane and Women's Hospital, Brisbane, Australia. [10]Rheumatic Diseases Unit, Institute of Genetics and Molecular Medicine, University of Edinburgh, Edinburgh, UK. [11]Medical Research Council (MRC) Centre for Causal Analyses in Translational Epidemiology, University of Bristol, Bristol, UK. [12]Department of Medical and Molecular Genetics, Indiana University School of Medicine, Indianapolis, USA. [13]Cardiovascular Health Research Unit, University of Washington, Seattle, USA. [14]Department of Biostatistics, Boston University School of Public Health, Boston, USA. [15]Department of Human Genetics, University of Pittsburgh, Pittsburgh, PA, USA. [16]Department of Public Health and Primary Care, University of Cambridge, Cambridge, UK. [17]Department of Twin Research and Genetic Epidemiology, King's College London, London, UK. [18]Centre for Bone and Arthritis Research, Institute of Medicine, Sahlgrenska Academy, University of Gothenburg, Gothenburg, Sweden. [19]Australian Centre for Ecogenomics, University of Queensland, Brisbane, Australia. [20]Department of Medicine, The University of Hong Kong, Hong Kong, China. [21]Research Centre of Heart, Brain, Hormone and Healthy Aging, The University of Hong Kong, Hong Kong, China. [22]Department of Medicine, Division of Endocrinology, Diabetes and Nutrition, University of Maryland School of Medicine, Baltimore, MD, USA. [23]Department of Human Genetics, Lady Davis Institute, McGill University, Montreal, Canada. [24]School of Medicine and Pharmacology, University of Western Australia, Perth, Australia. [25]Department of Endocrinology and Diabetes, Sir Charles Gairdner Hospital, Perth, Australia. [26]Centre for Population Health Sciences, University of Edinburgh, Edinburgh, UK. [27]MRC Human Genetics Unit, MRC Institute of Genetics and Molecular Medicine at the University of Edinburgh, Edinburgh, UK. [28]Department of Clinical Physiology, Turku University Hospital, Turku, Finland. [29]Research Centre of Applied and Preventive Cardiovascular Medicine, University of Turku, Turku, Finland. [30]Department of Medical Rehabilitation, Oulu University Hospital and Institute of Health Sciences, Oulu, Finland. [31]Division of Public Health Sciences, Fred Hutchinson Cancer Research Center, Seattle, USA. [32]Icelandic Heart Association, Kopavogur, Iceland. [33]Faculty of Medicine, University of Iceland, Reykjavik, Iceland. [34]Osteoporosis and Bone Biology Program, Garvan Institute of Medical Research, Sydney, Australia. [35]Department of Medicine, University of New South Wales, Sydney, Australia. [36]Department of Endocrinology, St Vincents Hospital, Sydney, Australia. [37]Department of Orthopaedic Surgery, Medical School University of Thessalia, Larissa, Greece. [38]Translational Genomics Research Institute, Phoenix, USA. [39]Laboratory of Epidemiology, Demography, and Biometry, National Institute on Aging, Bethesda, MD, USA. [40]Department of Internal Medicine, Hospital del Mar, Instituto Municipal de Investigación Médica (IMIM), Red Temática de Investigación Cooperativa en Envejecimiento y Fragilidad (RETICEF), Universitat Autònoma de Barcelona (UAB), Barcelone, Spain. [41]Unité de recherche en génétique humaine et moléculaire, Centre de recherche du Centre hospitalier universitaire de Québec - Hôpital St-François-d'Assise (CHUQ/HSFA), Québec City, Canada. [42]Department of Public Health and Clinical Medicine, Umeå Unviersity, Umeå, Sweden. [43]Musculoskeletal Research Programme, Division of Applied Medicine, University of Aberdeen, Aberdeen, UK. [44]Department of Endocrinology and Internal Medicine, Aarhus University Hospital, Aarhus C, Denmark. [45]MRC Lifecourse Epidemiology Unit, University of Southampton, Southampton, UK. [46]Ufa Scientific Centre of Russian Academy of Sciences, Institute of Biochemistry and Genetics, Ufa, Russia. [47]Biological Department, Bashkir State University, Ufa, Russia. [48]Division of Endocrinology and

Metabolism, Asan Medical Center, University of Ulsan College of Medicine, Seoul, South Korea. [49]Department of Genetics and Biotechnology, Faculty of Biology, University of Athens, Athens, Greece. [50]Department of Biochemistry and Experimental Medicine, The Children's Memorial Health Institute, Warsaw, Poland. [51]Department of Food and Environmental Sciences, University of Helsinki, Helsinki, Finland. [52]Jockey Club Centre for Osteoporosis Care and Control, The Chinese University of Hong Kong, Hong Kong SAR, China. [53]Department of Internal Medicine, University of Florence, Florence, Italy. [54]Department of Clinical Biochemistry, University of Ljubljana, Ljubljana, Slovenia. [55]Department of Medical Genetics, University of British Columbia, Vancouver, Canada. [56]Department of Endocrinology, University Medical Center, Ljubljana, Slovenia. [57]Division of Preventive Medicine, Brigham and Women's Hospital, Boston, USA. [58]Department of Medicine, University of Cambridge, Cambridge, UK. [59]Department of Surgical and Perioperative Sciences, Umeå Unviersity, Umeå, Sweden. [60]Department of Cardiology, Leiden University Medical Center, Leiden, The Netherlands. [61]Department of Gerontology and Geriatrics, Leiden University Medical Center, Leiden, The Netherlands. [62]Department of Internal Medicine, Division of Endocrinology and Metabolism, Medical University Graz, Graz, Austria. [63]Department of Epidemiology and Biostatistics, Extramuraal Geneeskundig Onderzoek (EMGO) Institute for Health and Care Research, Vrije Universiteit (VU) University Medical Center, Amsterdam, The Netherlands. [64]Department of Medicine and Therapeutics, The Chinese University of Hong Kong, Hong Kong SAR, China. [65]Department of Genetics, University of Barcelona, Centro de Investigación Biomédica en Red de Enfermedades Raras (CIBERER), Institut de Biomedicina de la Universitat de Barcelona (IBUB), Barcelone, Spain. [66]Department of Pharmacology and Therapeutics, University College Cork, Cork, Ireland. [67]Department of Health Sciences, University of Jyväskylä, Jyväskylä, Finland. [68]Department of Orthopaedics and Traumatology, Kuopio University Hospital, Kuopio, Finland. [69]Center for Clinical and Basic Research (CCBR)-Synarc, Ballerup, Denmark. [70]Department of Nutrition and Dietetics, Harokopio University, Athens, Greece. [71]Robertson Center for Biostatistics, University of Glasgow, Glasgow, United Kingdom. [72]Department of Endocrinology, Odense University Hospital, Odense, Denmark. [73]Clinical Institute, University of Southern Denmark, Odense, Denmark. [74]Department of Medicine, McGill University, Montreal, Canada. [75]Department of Medicine, University of Cantabria, Santander, Spain. [76]Department of Internal Medicine, Hospital Universitario Marqués de Valdecilla and Instituto de Formación e Investigación Marqués de Valdecilla (IFIMAV), Santander, Spain. [77]Department of Clinical Physiology, Tampere University Hospital, Tampere, Finland. [78]Department of Clinical Physiology, University of Tampere School of Medicine, Tampere, Finland. [79]Clinical and Molecular Osteoporosis Research Unit, Department of Clinical Sciences and Department of Orthopaedics, Lund University, Malmö, Sweden. [80]Department of Internal Medicine, University of Manitoba, Winnipeg, Canada. [81]Department of Endocrinology, Vrije Universiteit (VU) University Medical Center, Amsterdam, The Netherlands. [82]Extramuraal Geneeskundig Onderzoek (EMGO) Institute for Health and Care Research, Vrije Universiteit (VU) University Medical Center, Amsterdam, The Netherlands. [83]Department of Medical Sciences, University of Uppsala, Uppsala, Sweden. [84]Department of Pharmacology and Neuroscience, Umeå University, Umeå, Sweden. [85]Harvard Medical School, Boston, USA. [86]Department of Molecular Biology, Medical Biochemistry and Pathology, Université Laval, Québec City, Canada. [87]The APOGEE-Net/CanGèneTest Network on Genetic Health Services and Policy, Université Laval, Québec City, Canada. [88]Department of Molecular Epidemiology, Leiden University Medical Center, Leiden, The Netherlands. [89]Department of Chemical Pathology, The Chinese University of Hong Kong, Hong Kong SAR, China. [90]Li Ka Shing Institute of Health Sciences, The Chinese University of Hong Kong, Hong Kong SAR, China. [91]Department of Medical Genetics, University of Antwerp, Antwerp, Belgium. [92]Department of Medicine, Turku University Hospital, Turku, Finland.

[93]Department of Medicine, University of Turku, Turku, Finland. [94]Department of Legal Medicine, University of Cantabria, Santander, Spain. [95]Department of Human Genetics, McGill University, Montreal, Canada. [96]McGill University and Genome Québec Innovation Centre, Montreal, Canada. [97]Wellcome Trust Sanger Institute, Hinxton, UK. [98]Department of Orthopedic Surgery, Akureyri Hospital, Akureyri, Iceland. [99]Institution of Health Science, University Of Akureyri, Akureyri, Iceland. [100]Department of Epidemiology and Biostatistics, Lady Davis Institute, McGill University, Montreal, Canada. [101]Department of Medical Biochemistry, Oslo University Hospital, Oslo, Norway. [102]Medical Genetics Institute, Cedars-Sinai Medical Center, Los Angeles, USA. [103]Department of Endocrinology and Metabolism, University Hospital, Reykjavik, Iceland. [104]Genetics of Complex Traits, Peninsula College of Medicine and Dentistry, University of Exeter, Exeter, England. [105]Department of Clinical Biochemistry, Lovisenberg Deacon Hospital, Oslo, Norway. [106]Institute of Basic Medical Sciences, University of Oslo, Oslo, Norway. [107]Department of Medical Genetics, McGill University Health Centre, Montreal, Canada. [108]Division of Genetics and Rheumatology, Brigham and Women's Hospital, Harvard Medical School, Boston, United States. [109]Program in Medical and Population Genetics, Broad Institute, Cambridge, United States. [110]Department of Epidemiology, University of Pittsburgh, Pittsburgh, USA. [111]California Pacific Medical Center, San Francisco, CA, USA. [112]National Institute for Health and Research (NIHR) Musculoskeletal Biomedical Research Unit, University of Sheffield, Sheffield, UK. [113]Department of Internal Medicine, The Ohio State University, Columbus, USA. [114]Center for Clinical and Translational Science, The Ohio State University, Columbus, USA. [115]Menzies Research Institute, University of Tasmania, Hobart, Australia. [116]Durrer Center for Cardiogenetic Research, Amsterdam, The Netherlands. [117]Interuniversity Cardiology Institute of the Netherlands, Utrecht, The Netherlands. [118]Department of Clinical Chemistry, Tampere University Hospital, Tampere, Finland. [119]Department of Clinical Chemistry, University of Tampere School of Medicine, Tampere, Finland. [120]Center for Human Genomics, Wake Forest University School of Medicine, Winston-Salem, NC, USA. [121]Academic Unit of Bone Metabolism, Metabolic Bone Centre, University of Sheffield, Sheffield, UK. [122]Rural Clinical School, The University of Queensland, Toowoomba, Australia. [123]Department of Clinical Genetics, Erasmus Medical Center, Rotterdam, The Netherlands. [124]Department of Medicine, Indiana University School of Medicine, Indianapolis, USA. [125]Department of Medicine, University of Auckland, Auckland, New Zealand. [126]Department of Medicine, University of Davis, Sacramento, CA, USA. [127]Kolling Institute, Royal North Shore Hospital, University of Sydney, Sydney, Australia. [128]Department of Psychiatry, The University of Hong Kong, Hong Kong, China. [129]Centre for Reproduction, Development and Growth, The University of Hong Kong, Hong Kong, China. [130]Geriatric Research and Education Clinical Center (GRECC), Veterans Administration Medical Center, Baltimore, MD, USA. [131]Department of Preventive Medicine, University of Tennessee College of Medicine, Memphis, TN, USA. [132]MRC Epidemiology Unit Box 285, Medical Research Council, Cambridge, UK. [133]Framingham Heart Study, Framingham, USA. [134]Departments of Medicine, Epidemiology and Health Services, University of Washington, Seattle, USA. [135]Group Health Research Institute, Group Health Cooperative, Seattle, USA. [136]Medicine box 157, University of Cambridge, Cambridge, UK. [137]Departments of Medicine, Human Genetics, Epidemiology and Biostatistics, Lady Davis Institute, McGill University, Montreal, Canada. [138]Stanford Prevention Research Center, Stanford University, Stanford, USA.
